# Image Analysis Technique for Material Behavior Evaluation in Civil Structures

**DOI:** 10.3390/ma10070770

**Published:** 2017-07-08

**Authors:** Emanuela Speranzini, Roberto Marsili, Michele Moretti, Gianluca Rossi

**Affiliations:** Department of Engineering, University of Perugia, via Duranti, 93-06125 Perugia, Italy; roberto.marsili@unipg.it (R.M.); michele.moretti@unipg.it (M.M.); gianluca.rossi@unipg.it (G.R.)

**Keywords:** deformation, displacement, marker tracking, digital image correlation, no-contact measurement, monitoring, strain field, civil structures, masonry

## Abstract

The article presents a hybrid monitoring technique for the measurement of the deformation field. The goal is to obtain information about crack propagation in existing structures, for the purpose of monitoring their state of health. The measurement technique is based on the capture and analysis of a digital image set. Special markers were used on the surface of the structures that can be removed without damaging existing structures as the historical masonry. The digital image analysis was done using software specifically designed in Matlab to follow the tracking of the markers and determine the evolution of the deformation state. The method can be used in any type of structure but is particularly suitable when it is necessary not to damage the surface of structures. A series of experiments carried out on masonry walls of the Oliverian Museum (Pesaro, Italy) and Palazzo Silvi (Perugia, Italy) have allowed the validation of the procedure elaborated by comparing the results with those derived from traditional measuring techniques.

## 1. Introduction

The measurement of displacements has always been a necessity of structural mechanics, because in most structural systems, when appropriately formulated this quantity makes it possible to determine deformations and tensions. Research in the second half of the last century allowed the development of photoelastic methods based on the birefringence property possessed by some transparent materials (natural and artificial) when stressed by external forces [1]. The photoelastic investigation provides an overall image of the distribution of stresses, makes it possible to fully determine the stress regime in a loaded structure [2], to locate the concentration zones of the stresses due to the presence of a stress intensification factor where there is a defect or incision and to identify their direction [3]. Transmission photoelasticity has been successfully used for the study of granular materials such as elliptical particles in [4] but in rare cases it has been applied to the analysis of the stress state in masonry walls. In the interesting study described in [5,6], transmission photoelasticity was performed on scale models to evaluate the stress distribution in dry masonry walls made from brick with unilateral joints.

More recently, thanks to the evolution of digital image acquisition systems and the enormous growth of computing capabilities, research techniques based on image analysis have been developed, such as the numerical correlation of digital images [7], called digital image correlation (DIC). The DIC technique is based on the analysis of the differences between two images of an element under load, acquired at different moments [8]. The digital image is represented by an array of pixels, the color (or gray level) of each pixel is associated with a number representing the intensity. The analysis is based on the search for the maximum correlation between pixel intensities within the subsets of the image. Once the maximum correlation is identified, the displacement is known immediately from the subset being examined. This technique has developed rapidly, due to the rapid improvements in the quality and performance of the optics and in automated computing systems, and thanks to its usability and versatility of application.

The technique immediately found application in all areas of structural mechanics, enabling the evaluation of the deformation state of any type of structure and material in a non-destructive and non-contact manner. It was used for investigations of traditional structures [9,10,11,12], for example to study the fracture process of concrete structures [13,14], and to monitor the damaging of reinforced glass structures [15]. The technique becomes particularly interesting when combined with other investigative methods such as thermoelastic stress analysis, as it allows one to have full knowledge of the state of the material, both in terms of stress and strain, fundamental information both in the stages of product development and in testing and monitoring [16].

DIC has been used recently to evaluate the performance of innovative materials, for example to follow the deformation state of a carbon fabric reinforced cementitious matrix composite impregnated in situ [17], to investigate the validity of the bonding of fiber reinforced polymer [18,19], and to evaluate the effect of wet-dry cycles on the bond behaviour of concrete elements strengthened with NSM CFRP laminate strips [20]. As it is especially suitable for detecting deformation concentration zones [21], the technique has also been used in glass structures for locating defects, a particularly important problem given that cracks open up even for stresses much lower than the tensile strength of the material [15,22]. In contact areas between two surfaces, stress peaks are often generated, especially in the case of dynamic loads [23], which are difficult to diagnose with techniques such as thermoelastic stress analysis [24], as the optical access in the critical areas through windows transparent to infrared is certainly much more complex than the making of optical windows transparent to radiation in the range of visible wavelengths, for the use of DIC.

The use of the DIC technique is instead rather limited for masonry structures, which are generally tested using traditional, and sometimes destructive, tests and techniques. In this context, the first application of this technique involved the use of DIC in the laboratory to monitor masonry columns subject to axial eccentric loads [25], for the experimental analysis of concrete brick masonry-infilled frames [26], and to measure deformation fields in confined masonry walls built with hollow concrete bricks and surrounded by a reinforced concrete frame employing the three-dimensional digital image correlation [27].

Since 2008, the authors of this research have devoted themselves to the study of innovative monitoring techniques for historical structures, such as the use of fiber optics for monitoring [21,28] and the use of DIC for masonry structures with the goal of identifying the opening of cracks when they are still in the initial state (not visible to the human eye) and to monitor their evolution over time [22,29].

Having chosen to use this optical technique for historical masonry, one disadvantage is the need to create a speckle on the surface of the object, i.e., a random distribution, with high contrast and well-distributed, of small spots on the area being measured. In view of the invasiveness of this, the speckle has been substituted with markers applied to the masonry wall that can easily be removed at the end of the test, avoiding surface damage. The analysis of the structure under load must therefore follow the evolution of the markers to determine the displacement field. This method is identified by the term marker tracking (MT) and is based on the search and tracking of markers that move together with the masonry to which they are applied. The MT method is suitable for measuring the displacement field, but not for quantifying the evolution of the crack, for which DIC, with the use of adequate speckle, remains the best method.

For this purpose, special MATLAB software was developed in this study, which was then tested in a campaign of investigations of brick masonry samples specially made in the lab and of historical structures in situ. The deformation fields obtained were compared with the results of traditional measurement techniques, which allowed the validation of the process devised. 

## 2. Contactless Methods

The MT technique involves the acquisition of digital images of the details for which one wants to identify the displacement field following the application of a load or other events that cause it to arise. Two-dimensional applications are possible, where one can obtain the displacement field projected onto a normal plane at the optical axis of the camera, or 3D applications where one can have the 3D displacement field. For 2D applications a single camera is used, while for 3D applications, at least one pair of video cameras suitably positioned and fixed with regard to each other is used. The use of a pair of cameras translates into what in jargon is called stereovision, which allows the identification of the depth. Approaches of this type are used for the 3D reconstruction of object surfaces by means of the projection of structured light patterns [21,30], in which a large number of surface points are identified in order to reconstruct their geometry. MT differs from these geometric reconstruction systems in that its purpose is to obtain a field of displacements also for deformable bodies or portions of them belonging to the field framed. To achieve this, it is essential to be able to identify and follow the “signatures” on surfaces, i.e., well-defined areas that do not undergo variations of shape, size, and color during the test, even if the times can be very long, as in monitoring applications. To this end, it is common to use markers of known shape, size and color that are easily applied to the surface being investigated.

The MT method involves the acquisition of a series of images of the surface under investigation during the application of a load. The surface, undergoing a displacement field, pulls along with it the markers that are attached to it. The displacement field is then calculated only starting from the precise displacements of the individual markers, which if positioned with sufficient density, allow the calculation by interpolation of the displacement field. The use of high resolution cameras and low distortion lenses are necessary conditions for adequately capturing the investigated scene.

**Figure 1:**
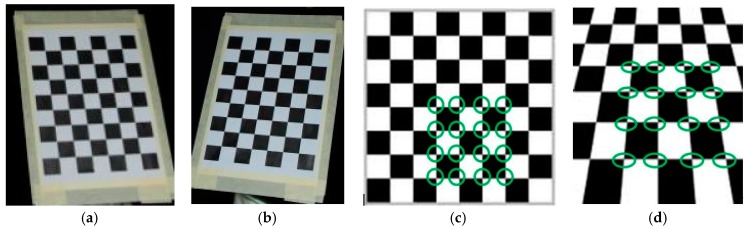
Stereo camera calibration checkerboard, (**a**) left (L) images. (**b**) right (R) images; (**c**,**d**) change in the pattern related to the perspective.

In this study, two Canon EOS 7D digital cameras are used, with an 18 megapixel sensor and 24 mm fixed focal length lenses, positioned on a system that keeps them fixed with respect to each other, creating what is commonly called a stereo camera. The use of commercial digital cameras has proved to be suitable, considering the purpose of the research, which was to develop and validate a low-cost method; in any event, the uncertainty of the measurement calculated was acceptable. When using cameras with better technical specifications, the metrological performance will obviously improve. In order to obtain 3D information, optical calibration techniques must be used, based on the simultaneous acquisition of specific targets. Black and white checkers (Figure 1) are typically used. Each “crossing” between 4 elements is located within each single image by means of the coordinates. The coordinates in the pixel array of the same point captured by both cameras define a pair of two-dimensional coordinates (*L_i_r*, *L_i_c*) and (*R_i_r*, *R_i_c*), where *L* and *R* represent the left camera and right camera, the index “*i*” represents a progressive index of the collected points, and “*r*” and “*c*” represent the row and column coordinates respectively within the pixel array. Acquiring a sufficiently large number of pairs of points of which the reciprocal position is known (the points on the checkerboard are coplanar and at a known distance from each other) makes it possible to write a function that links any pair of coordinates of the pixel array to a single 3D coordinate, according to the following type of function:

(1) $$\left[x,y,z\right]=f\left(\left(L_{i}r,L_{i}c\right),\left(R_{i}r,R_{i}c\right),s,PL,PR\right)$$

where the *x*, *y*, *z* values represent coordinates in space, s is a scale factor, and *PLrc* and *PRrc* are the coordinates of a generic point in the scene for which one wants to know the coordinates in space.

Equation (1) is characteristic of the stereo camera system and does not change until the lenses, the reciprocal position between the cameras or the cameras themselves are changed. This means that once the calibration procedure has been carried out, the stereo camera can be applied to any scene, and starting from the coordinates of the pixel array of both cameras of the same entity extent belonging to the scene, it is possible to obtain the spatial coordinates in the reference system integral with the stereo camera. The use of this technique is virtually extendible to a large number of cameras, that is, to a large number of shots (pairs of images at different times) from different points of view, making possible the complete 3D mathematical reconstruction of any object [29]. The space framed by the cameras becomes measurable, as do the positions of points and their reciprocal distances. By performing several acquisitions, it is possible to differentiate the positions reached by the various points in the scene, thus being able to define applied displacement vectors and speed vectors if the acquisition frame rate is known.

The system’s intrinsic insensitivity to the positioning once the calibration parameters are known make double-camera MT particularly suitable for discontinuous monitoring of displacements of existing structures, preferable to a single camera system, which requires the correct repositioning of the acquisition system at a later time. The single-image photogrammetric system is in fact subject to perspective phenomena that cannot be compensated for and that inevitably lead to the incorrect calculation of the markers’ positions. Considering the 4x4 pattern of Figure 1c and its transformation in Figure 1d, ignoring the variation due to a scale effect, it is evident that the points of the first line at the bottom have moved away from each other, while the points on the first line at the top are closer to each other. As regards the parallel vertical lines, these are changed to convergent at the vanishing point of the perspective image, decreasing their relative distance from the bottom toward the top. From these two images one obtains a non-null vectorial displacement field, but that is only and exclusively the result of a change in the perspective of the image, i.e., a different relative positioning between the framed object and the camera. From this example it can be seen how the two dimensional approach, though simpler, is not operationally reliable for measurements in discontinuous applications that involve the repositioning of the camera. The problem of perspective can be corrected, however, by making use of stereovision, because changing the position of the pair of video cameras changes the perspective of both cameras according to a mathematical function Equation (1), which can be used to determine the correct vector displacement field.

**Figure 2:**
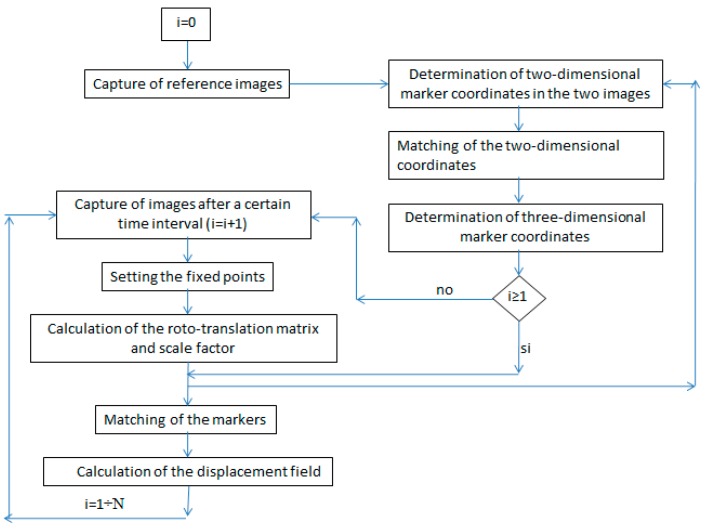
Flow-chart of the schematic representation of the algorithm.

For example, in two distinct acquisitions, the points (markers) are identified 3D, but it is highly unlikely that there is a perfect overlap of the marker positions in the pixel arrays, due to the repositioning of the camera and to the actual movement by the markers. However, landmark points considered fixed can be set within the images, and used to calculate a 3D roto-translation matrix that realigns the reference systems. With this correction approach, the 3D coordinates of the fixed points both in the reference acquisition and in the subsequent acquisitions remain unaltered, and the displacement field can be calculated by the differentiation of the positions of the markers within the image. The schematic representation of the algorithm is shown in the flowchart in Figure 2.

The experimental analyses carried out together with the bibliographic search made it possible to show how both the MT and DIC techniques can be applied for monitoring, with both having advantages and disadvantages in comparison with each other. Table 1 summarizes the main differences and the characteristics that led to the choice of working with the MT technique rather than with DIC. In light of the aforesaid factors, the marker tracking technique is suitable for the monitoring of existing masonry. The instrument must be portable and able to detect the evolution of the displacement field of the framed object quickly and over time, without having to reposition the instrument in precisely the same position. The instrument must also have an intrinsic sensitivity to brightness conditions. Lastly, there should preferably be zero preparation of the surfaces, or it should at least be noninvasive in the cases where it becomes necessary. Regarding the repositioning, the 3D MT technique does not suffer from this limit, as it allows for a rescaling of the images and if necessary, a correction of perspective. Another decisive factor is the surface preparation, which in the case of DIC consists of painting the surface to form a random distribution of white spots on a black background. MT can make use of high contrast areas on the surface, or small white and black concentric removable adhesive disks, typically used in reverse engineering scanning applications in the mechanical field, can be applied to the surface. The resolution that can be achieved by the tracking technique is of a lower order; however, it is the price that must be paid if one wishes to obtain a strong, reliable measurement technique. The variations in brightness influence the measurements with DIC, but in this work the variation of the light is not significant because the tests are of short duration and the lighting is artificial and controlled.

## 3. Results

### 3.1. The Software Developed in This Research

The software developed in this study is a hybrid MT-DIC type. More specifically, it makes use of marker tracking principles to find the position of the markers in the different images, and then uses image correlation to optimize the overlapping of the markers themselves, reaching subpixel resolutions with a magnification factor up to 10, in order to obtain acceptable computing time consuming. With this approach, one obtains the advantage of increasing the resolution of the method without significant computational increases.

**Figure 3:**
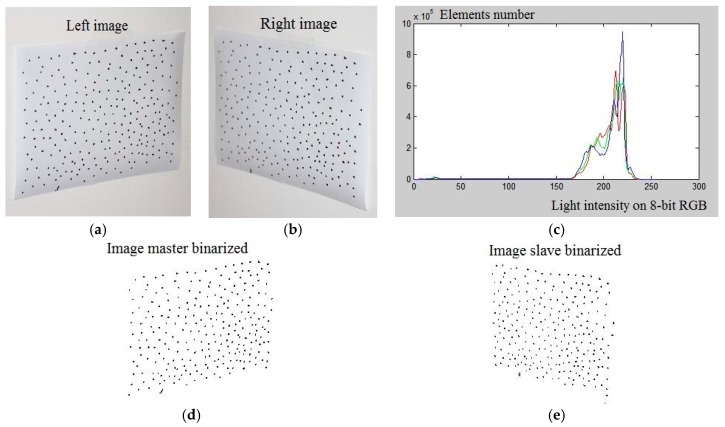
(**a**,**b**) Software, loading of the “right” and “left” images and their display. (**c**) image analysis, chromatic distribution and level filter, the curves represent the three colors RGB (red, green, blue). (**d**,**e**) Image binarized.

The software required for complete execution of the measurement uses the images acquired as input, and as output it provides a text file containing the 2D coordinates of the markers in the two images together with a set of statistical parameters that can characterize the geometric parameters of shape and size. The software loads the left and right images (Figure 3). For the implementation stage, a high-contrast pattern in A4 format is used. The use of this is very practical, as the target can be digitalized using a common 600 dpi scanner to obtain a reference datum for the validation of the algorithms implemented. Areas of varying shape and size are used as markers, with spacing that is random but sufficiently isolated to avoid mismatching problems. 

**Figure 4:**
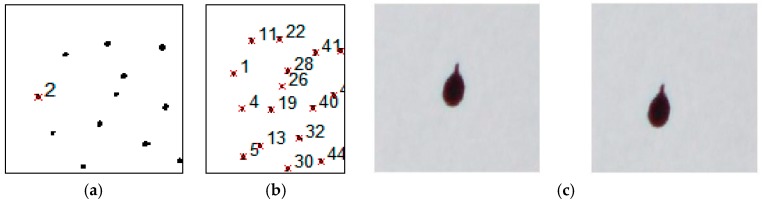
(**a**,**b**) Incongruent enumeration between left “a” and right “b” images. (**c**) Image regions acquired at different moments containing the same marker.

The two cameras were used for the acquisitions with a 24 mm fixed focal length high brightness lens, combined with two high-power flashes. The uncompressed capture mode is used to maintain the highest chromatic and contrast fidelity. The first step involves the binarization of the image, obtaining 1/0 logical maps. The high chromatic contrast highlights an easily segmented bimodal distribution that can be obtained by means of a threshold level around the intensity value 100 on an 8-bit RGB scale (Figure 3d,e). The markers in the individual image are then identified and labeled, characterized by means of the barycentric coordinates. The next step is checking the congruence of the enumeration of the markers, as the same marker could be identified with different numbers in the two images, as shown in Figure 4a,b.

The procedure studied is based on plane linear geometrical transformation algorithms that allows the matching of the markers in the two images. Unidentified markers in both images are discarded. This situation can be seen when dealing with curved surfaces where one or more markers may be beyond the horizon line with regard to a camera, or outside the field framed by one of the two cameras. The position in the 3D space integral to the stereocamera of each correctly identified marker is obtained by applying the transformation introduced schematically by Equation (1). By taking a second shot, which captures the evolution over time of the displacements, carrying out again the sequence of operations above, it is possible to recalculate the 3D positions of each individual marker. By indicating a group of three markers that are assumed not to have undergone relative displacements, the displacement field is calculated based on the positions reached by the remaining markers.

**Figure 5:**
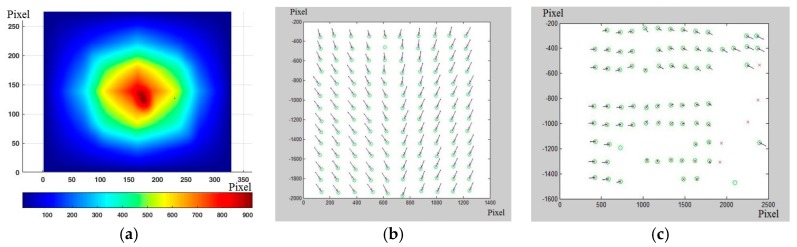
(**a**) Map of local cross correlation, from which the displacement vector is extracted; (**b**,**c**) Example of displacement applied vectors obtained by means of the method used.

Once the position of the barycenter of each individual marker in the image matrices (L/R) acquired at different times (T_0_, Ti) is known, it is possible to select the regions of each single image that contain the individual marker, as shown in the following in Figure 4c. In order to increase the method’s spatial resolution, a cross correlation is used, identifying the most accurate displacement even in the case of a partial modification of the marker morphology (Figure 5a). The coordinates of the maximum crosscorrelation value indicate the displacement value that maximizes the overlapping of the images that contain the single marker. The cross correlation value provides quantitative information on the morphological change of the marker, which if excessive is automatically discarded. Using cross correlation increases the resolution of the displacement by a factor of 10 and increases its accuracy. This procedure presupposes a limited perspective change and a limited morphological variation of the marker, as already indicated in the preceding paragraphs. Knowing the displacement vector allows the vectorial representation of the displacements, such as those shown in Figure 5b,c, for each pair of images captured after the reference images.

**Figure 6:**
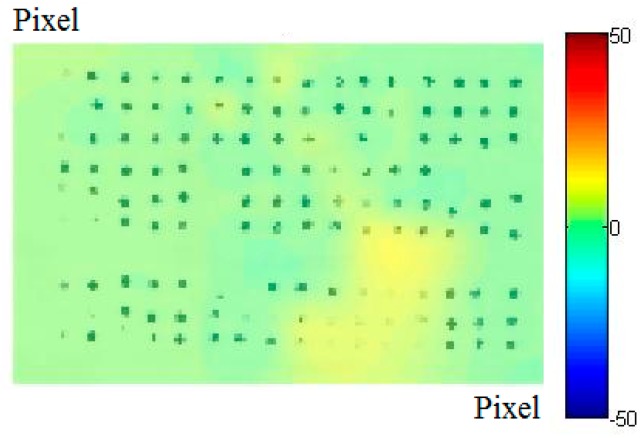
Displacement field.

From these, through interpolation it is possible to obtain the displacement field, which can be represented as an image overlapping the reference images (Figure 6).

### 3.2. Experiments on Masonry Structures for the Validation of the Investigation Methodology and Results

The research methodology and the related software were validated by experimenting with existing masonry structures, typical of many historical town centers, with the aim of comparing the results obtained with the methodology proposed here with others deriving from technologies and methods of proven validity [31,32,33,34,35]. The first test was carried out in the laboratory on specially-built masonry samples, and subsequently other tests were carried out in situ on existing historic masonry.

#### 3.2.1. Laboratory Experiments on Masonry Samples 

**Figure 7:**
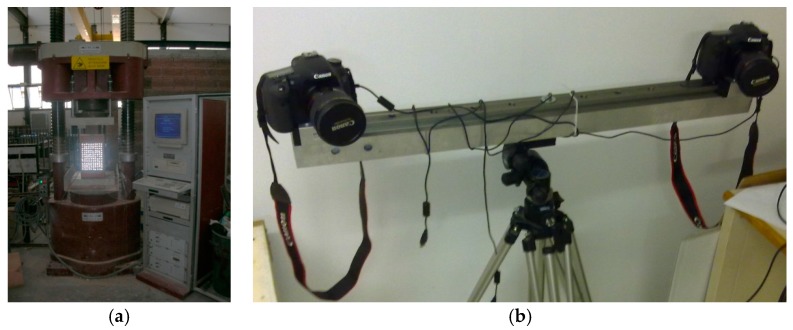
(**a**) Sample prepared with the markers and set up on the press for the compression test; (**b**) equipment for image acquisition.

The investigation consisted of a compression test on masonry samples that allowed a comparison of the structural response assessed in terms of displacements measured using traditional systems such as linear variable displacement transducers (LVDT) and using the method proposed here. Three masonry samples having dimensions of 240 mm × 240 mm × 400 mm were specially built for this purpose with solid bricks and premixed type M1 cement-lime mortar (strength 2.5 MPA) at the Department of Engineering of the University of Perugia (Terni campus). After they had cured the specimens were prepared for the compression test: for the measure of the displacements the specimens were instrumented with LVDT and prepared with markers in order to be able to acquire suitable images to be processed with the MT software developed specifically by us for monitoring masonry. In the latter, a fundamental step is the choice of the markers to be applied on the front face of the sample, which must be of a well-defined shape and provided with a very high brightness. In the software the marker is identified on the basis of the luminous intensity and in reference to its shape, in order to avoid that other surfaces that reflect similarly to the same (such as metallic surfaces) may influence the identification. Thus if the markers have a well-defined geometric shape, the software can automatically discard the elements having a different shape. In this experiment, markers with a circular shape and made of reflective material were chosen and applied so as to create an orderly pattern. The images were acquired a 24 mm fixed focal length and two flashes synchronized to provide constant and uniform lighting during the test, all mounted on a rigid tripod (Figure 7b). The instrumentation was connected to a computer that also manages the frequency of the image acquisition and stores them. At the same time, a “Metrocom”-type press was used to apply the monotone compression load, increasing until failure (Figure 7a). Briefly, during the test the load, the displacements measured with the LVDT transducers applied to the masonry samples, and the sequence of images for reprocessing with the proposed technique were recorded.

**Figure 8:**
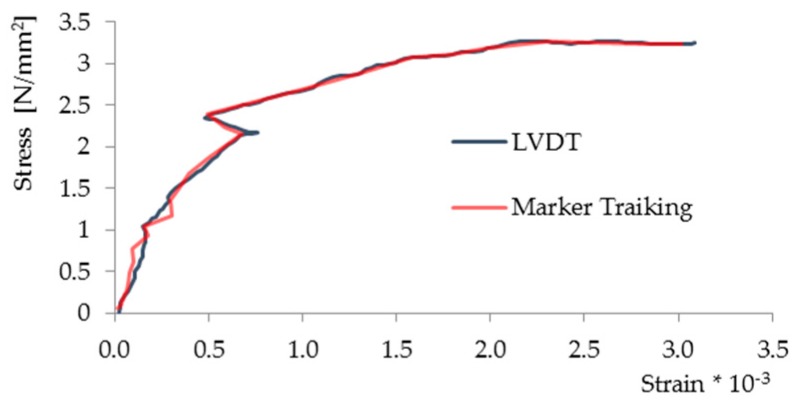
Displacement measurement with linear variable displacement transducers (LVDT) and the proposed methodology (MT).

The processing, done with the software developed, begins with the identification of the markers based on shape and on the value of the luminous intensity, which the element acquires when it is illuminated by the flash (Figure 7b). The barycenter coordinates are determined from the contour of the marker, and the displacement is calculated by recognizing the same marker in two sets of subsequent images. During this stage it is possible that not all markers are recognized: the main causes can be traced to the absence of the marker in subsequent photos (the marker may have become detached) or to the fact that the motion brings one or more markers outside the visual field of at least one of the two cameras. In this regard, it is important to emphasize the need to apply a sufficiently large number of markers so that the loss of some of them does not compromise the test. Once all the markers are recognized in both sets of photos, the displacements along the x, y and z axes of the vector field can be calculated. The processing of the results makes it possible to obtain the stress-strain diagrams of Figure 8, where strains were measured using the methodology proposed here and the LVDT displacement transducers, obviously referring, in both the measurement methods, to measurements from the same position. It can be seen from the comparison that there is a good correspondence between the two graphs: the maximum calculated uncertainty is 2.0% of the maximum measured displacement, considered as full scale value, and the coefficient of the linearity between the measurement from LVDT and MT is 0.998. It can be noted that the measurement made with LVDT consists of a high number of points acquired with a frame rate set by the acquisition system used, whereas the images were acquired with a lower frame rate. However, higher frame rates can be used for monitoring the observed phenomenon with greater temporal resolution.

#### 3.2.2. Experiments on Masonry In Situ

Other investigations with the proposed methodology were carried out in situ on historic buildings during masonry compression tests using the double flat jacks technique. In this type of test, a portion of the wall panel is subject to compression, in a normal direction to the brick laying plane; the load is generated by two flat jacks that are inserted inside the masonry at a mortar joint, after making a cut.

**Figure 9:**
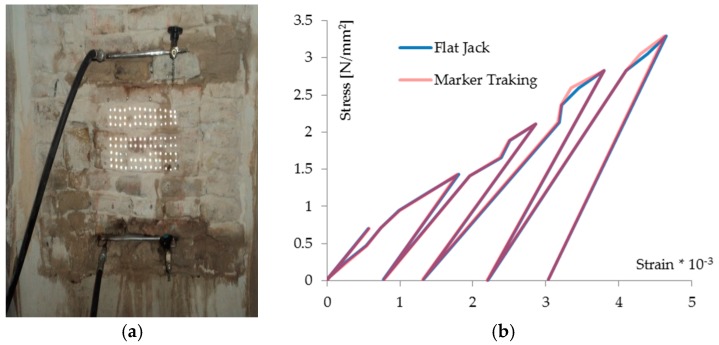
**(a**) The test on a masonry wall inside the Oliverian Museum of Pesaro, (**b**) Stress-strain diagrams with the flat jack and the proposed technique (MT).

The behavior of the masonry is detected by means of the measurement of the deformations using a deformometer, and thus for this purpose the masonry wall is prepared with the application of measurement bases necessary for the measurement of the displacements. During the test, the pressure applied and displacement values measured with the deformometer are recorded, and are then processed to obtain the deformations in the direction parallel to the applied load. In addition, a set of markers for evaluating the deformations using the proposed technique were prepared on the surface of the masonry in these tests. The first case presented here concerns the Oliverian Museum of Pesaro, a historic building in an Italian historic town center. The masonry panels were prepared for the test with flat jacks as usual, and subsequently the markers (shiny steel circles, diameter 5 mm) were applied that were to be used for the proposed technique (Figure 9a). 

The illumination by means of flash gives the image a high contrast between the marker and the background, a necessary condition for the correct application of the method. The compression test, carried out for load cycles, provided the results shown in Figure 9b, which gives the stress-strain diagrams resulting from the two techniques. Their comparison shows a very good correspondence between the curves of the two diagrams, and this demonstrates the validity of the proposed methodology for the measurement of deformations in masonry structures.

**Figure 10:**
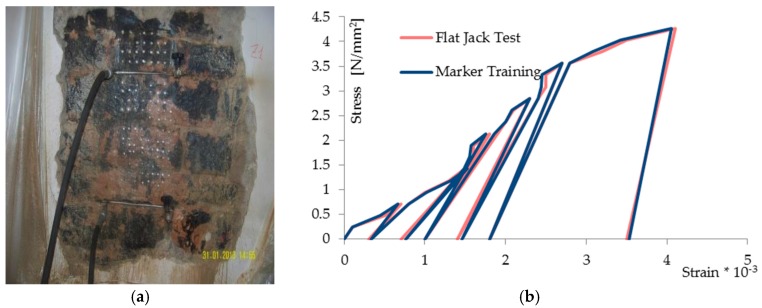
(**a**) Palazzo Silvi: wall prepared for the Flat Jacks and Marker Tracking tests; (**b**) The stress-strain diagrams obtained from the cyclic load test for the two techniques.

**Figure 11:**
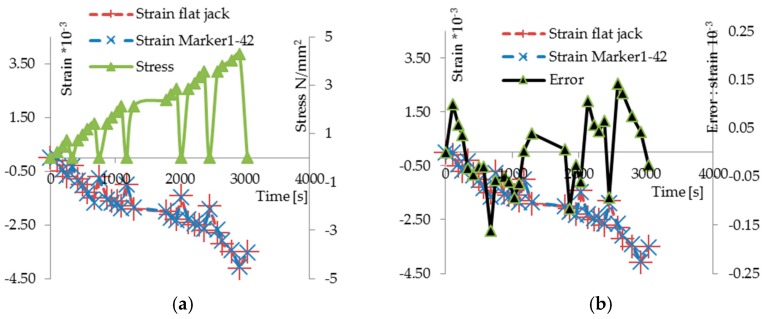
(**a**) Stress/strain versus time: stress (right axis), strain (left axis); (**b**) Measurements with MT (blu line) e deformometer (red line). Black line shows the values of the differences between two measurements (scale in right axis: strain 10^−3^).

An investigation similar to the previous one was carried out for the compression tests with double flat jacks at Palazzo Silvi in the historic center of Perugia, again in order to compare the results of the MT technique with those obtained from traditional techniques. Figure 10a shows the masonry surface prepared for the tests: the measurement bases for the evaluation of the deformations by means of the flat jacks test and the markers for the proposed technique were applied. The photo also shows the two jacks inserted into the mortar joints (one upper and the other lower) connected to the pump. The results are shown in the stress-strain diagrams of Figure 10b. In addition, the diagram in Figure 11a shows the results in reference to a pair of markers. It gives: the time on the abscissa axis, the stress on the right axis of ordinates, and the strain measured with the deformometer (red line) and with the proposed method (blue line) on the left axis of ordinates. Referring to the measurements made with the deformometer, the diagram in Figure 11b allows us to evaluate the relative uncertainty of the results of the new methodology compared with those obtained with the deformometer. The uncertainty measured on tests in situ show a good overlapping of the two measurements; the calculated maximum absolute uncertainty is 14 × 10^−3^ strain. In this research, a metrological characterization of the full scale uncertainty is not presented because the proposed methodology has virtually an infinite full scale. Assuming in this test, the measured maximum value by deformometer of 4.058 × 10^−3^ strain, as full scale, the maxim absolute uncertainty is 3.5%, which is only significant of this test. 

## 4. Reliability of the Measurements and Discussion

The comparison between the results obtained with the proposed method and those derived from traditional techniques demonstrates how the former can be used effectively in the field of displacement measurements, offering benefits with regard to the speed of execution of the measurement and the safety of the measurement, as the reading of the measurement by an operator is not necessary. The application of this method also allows us to have a considerable amount of information available on the evolution of the deformation phenomenon, being able to associate a displacement vector with each moment and each marker. As is shown by these, it is possible to obtain displacement fields by interpolating the results from the markers, allowing one to identify any irregularities in the field due to problems or characteristics of the masonry considered.

## 5. Conclusions

The methodology proposed for structural monitoring is based on the capture over time of a series of images of the surface of the structure and the analysis of the correlation of those images. For this purpose, a MATLAB-based software was developed that uses the images acquired to evaluate the state of deformation of significant points on the surface of the structure following the evolution of the displacement of markers applied to it. The output is the text file containing the coordinates of the markers in the two images together with a set of statistical parameters that can characterize geometric parameters of the surface’s shape and size. With this approach, subpixel resolutions can be achieved with a magnification factor of 10 that increases the resolution of the method without significant computational increases. 

The validity of the methodology presented was verified by performing tests on masonry subjected to compression and measuring the corresponding state of deformation with both the proposed methodology and other traditional techniques of proven validity at the same time.

Laboratory compression tests on specially-built masonry sample sand in situ tests with double flat jacks on two different historical buildings have provided results comparable to those obtained using traditional techniques, proving the validity of the method as regards the measurements.

Moreover, the proposed methodology has the advantage of being easy to use on existing structures. The use of easily removable markers makes the technique noninvasive, and thus it can also be used for periodic monitoring, with it being possible to reposition the instrumentation without introducing uncertainty.

## Figures and Tables

**Table 1:** Comparison between the characteristics of the marker tracking (MT) (2D, 3D) and digital image correlation (DIC) (2D, 3D) techniques.

Characteristic	Marker Tracking		Digital Image Correlation	
Resolution	1 pixel		1/100 pixel	
Surface preparation	High contrast target, stickers		High contrast speckle, area of interest must be painted	
Sensitivity to environmental variables, light	Poor		High	
Sensitivity to repositioning	2D	Very high (it is not possible to correct changes in perspective	2D	Very high (it is not possible to correct changes in perspective
	3D	Poor (the stereo camera can be “recalibrated” provided that the angle of perspective does not change too much)	3D	Poor (the stereo camera can be “recalibrated” provided that the angle of perspective does not change too much)
Calculation time	Very rapid calculation		Calculation speed depends on the desired spatial resolution	
